# A comparison of the effect of two methods of positioning the hands during basic and advanced cardiovascular life support on the chest compression depth in adults

**DOI:** 10.15171/jcvtr.2019.51

**Published:** 2019-10-06

**Authors:** Mohammadreza Bastami, Parand Soliemanifard, Roholla Hemmati, Golnaz Forough Ameri, Mahboobeh Rasouli, Masoumeh Shohani

**Affiliations:** ^1^Department of Nursing, Faculty of Nursing and Midwifery, Ilam University of Medical Sciences, Ilam, Iran; ^2^Department of Cardiology, Faculty of Medicine, Ilam University of Medical Sciences, Ilam, Iran; ^3^Department of Public Health Nursing, Razi Faculty of Nursing and Midwifery, Kerman University of Medical Sciences, Kerman, Iran; ^4^Department of Biostatics, Public Health School, Iran University of Medical Sciences, Tehran, Iran

**Keywords:** ACLS, BCLS, Chest Compression, Manikin

## Abstract

***Introduction:*** There is no agreement on how the hands are positioned in cardiopulmonary resuscitation (CPR). In this study, the effects of two methods of positioning the hands during basic and advanced cardiovascular life support on the chest compression depth are compared.

*** Methods:*** In this observational simulation, the samples included 62 nursing students and emergency medicine students trained in CPR. Each student performed two interventions in both basic and advanced situations on manikins and two positions of dominant hand on non-dominant hand, and vice versa, within four weeks. At each compression, the chest compression depth was numerically expressed in centimeter. Each student was assessed individually and without feedback.

*** Results:*** The highest mean chest compression depth was related to Basic Cardiovascular Life Support (BCLS) and the position of the dominant hand on non-dominant hand (5.50 ± 0.6) and (*P* = 0.04). There was no statistically significant difference in the basic and advanced regression variables in men and women except in the case of Advanced Cardiovascular Life Support (ACLS) with dominant hand on non-dominant hand (*P* = 0.018). There was no significant difference in mean chest compression during basic and advanced cardiovascular life support in left- and right-handed individuals (*P* = 0.09).

***Conclusion:*** When the dominant hand is on the non-dominant hand, more pressure with greater depth is applied.

## Introduction

Timely performance of a cardiopulmonary resuscitation (CPR) rescuer is one of the key factors in increasing the survival rate in victims of OHCA, but nevertheless only a small number of victims of OHCA are potentially saved by the CPR rescuer. In this regard, CPR training may help improve the outcomes of cardiac arrest.^1^

Over the past decade, devices have been developed that offer audio and video feedback during compression of the chest to improve the quality of the CPR.^2,3^ However, whether these methods and devices increase chest compression efficiency is controversial.^4,5^ An alternative approach to improve the performance of rescuer was placing the dominant hand against the chest during compression.^6^ In this regard, Nikandish et al^7^ reported that the quality of chest compression within 5 minutes was in accordance with previous guidelines for chest compression CPR independent of the hand in contact with the chest. Kundra et al^8^ also reported that compression of the chest was done with fewer mistakes when the dominant hand contacted the chest.

In the protocol proposed by the American Heart Association, the fact that the dominant hand must be positioned on the non-dominant hand for compression or vice versa is not mentioned. It seems that the chest compression depth is different in the two cases. In this study, the effects of two methods of positioning the hands during basic and advanced cardiovascular life support on the chest compression depth are compared.

## Materials and Methods

In this single-blind interventional study, the statistical population included 62 nursing students and emergency medicine students, who entered the study through census. The inclusion criteria were nursing students of semesters 6 and 8, and emergency medicine students of semester 3 who completed basic and advanced cardiovascular life support training courses. Students’ unwillingness to participate in the study or continuing their participation were considered as exclusion criteria. The study environment was Clinical Skills Center at the Faculty of Nursing and Midwifery of Ilam University of Medical Sciences. The study was conducted from November 2017 until April 2018.

The students reviewed the basic and advanced cardiovascular life support workshop one week before the intervention for four hours under the supervision of the trainer, and then each student performed four interventions (two interventions in the two basic and advanced situations). In the first intervention, the student was asked to resuscitate the patient on the ground (basic cardiovascular life support) assuming that the patient’s airways have been stabilized, while the dominant hand was positioned on the non-dominant hand for chest compression. Each time, only 3 compressions were applied on the chest and at each respiratory cycle, 3 breaths were given.

In the second step, the student was asked to resuscitate the patient on the ground (basic cardiovascular life support), while the non-dominant hand was positioned on the dominant hand for chest compression. Each time, 3 compressions were applied on the chest and at each respiratory cycle, 3 breaths were given.

In the third step, the student was asked to complete the first and second steps on the manikin (JYCPR-007 Half Body CPR Training Manikin), which was located on the resuscitation bed (advanced cardiovascular life support).

Giving breath was measured by manikin but was not recorded. In fact, the purpose of giving breath was conducting a single-blind study. In addition, the students were told that the purpose of the study was to “assess the students’ knowledge of cardiopulmonary resuscitation”.

Since tiredness can affect the chest compression depth, each student gave his/her place to another student after compressing the chest, and did not perform resuscitation until the end of the list. One week after the resuscitation by all students, the second intervention took place from the beginning and in the same order. It should be noted that each student was assigned a number as a code to maintain order.

The correctness or incorrectness of the position of the hands was determined by the Manikin. For each compression, the Manikin confirmed the correctness or incorrectness of the compressed location, and the intervention was repeated in the following days if the compression position was not correct.

Each student was asked to compress the Manikin chest 3 times with maximum force in each intervention. At each compression, the obtained number was recorded by the researcher. The final number, which indicates the compression force, was the average of these 3 numbers.

In the third and fourth interventions, which took place during the third and fourth weeks after the first intervention, the above interventions were repeated. However, the Manikin was placed on the standard resuscitation bed this time; resuscitation on the bed was considered as Advanced Cardiovascular Life Support (ACLS) and resuscitation on the ground as Basic Cardiovascular Life Support (BCLS).

At each compression, the Manikin showed the chest compression force numerically in centimeter. The higher the number, the higher the chest compression force. This number was recorded by the researcher. The student could not see the Manikin display and the record sheet.

All students were on the right side of the manikin for chest compression. In fact in this study, no matter what the dominant hand was, the dominant hand of all participants was once considered as the right hand and once the left hand in a crisscross form. Each student was also assessed individually, but they were not provided with any feedback. It should be noted that the correctness of the massage performed by the students is confirmed by manikin and coach.

SPSS version 21 was used to analyze the data. The significance level of data was considered to be *P* <0.05.

## Results

Overall, 62 students participated in the study (Table 1). The mean and standard deviation of the depth of chest compression in centimeter in both basic (BCLS) and advanced (ACLS) modes and how the hands are positioned together in resuscitation are presented in Table 2. The results show that the lowest mean is related to BCLS and the position of the non-dominant hand on the dominant hand (4.22 ± 1.8). In addition, the highest mean was related to BCLS and the position of the dominant hand on the non-dominant hand (5.50 ± 0.6).

**Table 1:** Demographics characteristics of participants

**Variable**		**No.**	**%**
Semester	6 Nursing	21	34
	8 Nursing	23	37
	3 Emergency medicine	18	29
Gender	Male	46	74
	Female	16	26
Height	Male	Men (181.15)	SD (5.7)
	Female	Men (161.94)	SD (4.5)
Weight	Male	Men (79.3)	SD (13.9)
	Female	Men (59.44)	SD (5.3)
Age	Max. (45), Min. (21)	Men (25.03)	SD (5.4)

**Table 2:** Mean and standard deviation of the depth of chest compression in centimeter in basic and advanced cardiovascular life support

**Resuscitation technique**	**Hand**	**N**	**Minimum**	**Maximum**	**Mean (depth/CM)**	**Standard Deviation**
BCLS	DH	62	4.00	6.00	5.5000	0.60055
	NDH	62	0.00	6.00	4.2258	1.97873
ACLS	DH	62	2.00	6.00	4.9903	0.77517
	NDH	62	1.00	6.00	4.4452	0.85271

Abbreviation: DH, dominant hand; NH, non-dominant hand; BCLS, Basic Cardiovascular Life Support; ACLS, Advanced Cardiovascular Life Support.

The results of Mann–Whitney U showed that there was no statistically significant difference in variables between men and women, except for the advanced cardiovascular life support and the position of dominant hand on non-dominant hand (*P* = 0.018) and in terms of dominant hand showed that there was no significant difference between the two cases regarding dominant and non-dominant hands and the dominance of the right hand (*P* > 0.05).

In addition, there was no significant difference in mean chest compression depth in basic and advanced cardiovascular life support in left- and right-handed individuals (*P* = 0.09).

Linear correlation shows that there is no significant relationship between depth of chest compression and height and weight in basic and advanced cardiovascular life support when dominant hand is positioned on non-dominant hand. Moreover, in other resuscitation cases, despite a significant relationship between depth of chest compression and height and weight, linear correlation coefficient was in the range of 0.1 to 0.3, which indicates a linear relationship with weak intensity between variables. Furthermore, despite a significant correlation (*P* = 0.018) between advanced cardiovascular life support with non-dominant hand on dominant hand and weight, the linear correlation coefficient was in the range of 0.299, indicating a linear relationship with weak intensity between variables.

## Discussion

The results of this single-blind study showed that in basic and advanced cardiovascular life support, the way the hands are positioned and which hand is in contact with the chest affect the depth of chest compression; the depth of chest compression is greater if the non-dominant hand is in contact with the chest. However, the mean depth of chest compression in basic cardiovascular life support was higher than advanced cardiovascular life support. There is no recommendation in this regard in the European (ERC) and American guidelines (AHA),^8,9^ and the results of different studies contradict each other.

A study by Nikandish et al^7^ showed that placement of the dominant hand on the chest increased the number of correct external chest compressions (ECCs) in the participants, but no statistically significant relationship was observed. These results were similar for men and women. However, the results of a study by Kundra et al^10^ showed that the placement of dominant hand on the chest significantly increased the number of correct ECCs in the participants.

Owen et al^11^ compared the effectiveness of chest compression based on hand position and the number of chest compressions based on the two 2005 and 2000 ERC guidelines. The results of their clinical trial indicated that the mean chest compression in the educational group with the 2005 guideline was 102, while it was 104 in the educational group with the 2000 guideline. The number of cases of incorrect hand position in the 2005 group was 24, while it was 9 in the 2000 group.

According to the study of Owen et al, techniques of measurement index regarding hand position for ECC do not have negative effects on the number of chest compressions during the basic life support and improves the correctness of hand position.^11^

Differences between the results of this study and other studies may be due to differences in experiences of participants (students in this study, rescuers^7^ and anesthesia residents,^10^ study method and manikins used in resuscitation: UCC-CPR versus standard chest compression-ventilation CPR^10^ and JYCPR-007 Half Body CPR Training Manikin in this study), and the difference in sample size.

In this study, there was no statistically significant relationship between the depth of chest compression and the dominant hand. In other words, right-handedness or left-handedness did not affect the depth of chest compression during resuscitation. However, Jo et al^12^ suggest that ECC in the group that used dominant hand was significantly faster than the group that used non-dominant hand. The contact of the dominant hand with the chest can affect the depth of chest compression.

## Limitations

One of the limitations of this study is the use of manikins. Performing CPR by a simulated scenario cannot adequately provide cases of chest compression and physiological differences among victims of cardiac arrest.

## Conclusion

The results of this study show that when the dominant hand is on the non-dominant hand, it applies more pressure than when it is positioned in the opposite direction. Simply put, in CPR, which emphasizes the effective chest compression, it is better to put the dominant hand on the non-dominant hand to activate blood circulation efficiently.

## Competing interests

None.

## Ethical approval

The ethical approval of the project (ir.medilam.rec.1397.090) was obtained from the Ethics committee for Science and Technology of Ilam University of Medical Sciences.

## Acknowledgments

We sincerely appreciate Ilam University of Medical Sciences for their financial support. Also we appreciate students for their cooperation.

## Funding

This study was funded by Ilam University of Medical Sciences.
